# Analyses of adult transcriptomes from four different populations of the spongy moth, *Lymantria dispar* L., from China and the USA

**DOI:** 10.1038/s41598-022-18377-4

**Published:** 2022-10-29

**Authors:** Yi-Ming Wang, Michael E. Sparks, Robert L. Harrison, Juan Shi

**Affiliations:** 1 Sino-France Joint Laboratory for Invasive Forest Pests in Eurasia, Beijing Forestry University, Beijing, 100083 China; 2 Invasive Insect Biocontrol and Behavior Laboratory, USDA-ARS, Beltsville, MD 20705 USA

**Keywords:** Molecular biology, Zoology

## Abstract

The spongy moth *Lymantria dispar*, formerly known as the gypsy moth, is a forest pest that occurs as two different biotypes: the European spongy moth (ESM), *Lymantria dispar dispar*, which is distributed in Europe and North America; and the Asian spongy moth (ASM), which consists of subspecies *Lymantria dispar asiatica* and *Lymantria dispar japonica* and is distributed in China, Russia, Korea, and Japan. The Asian biotype is classified as a quarantine pest by the U.S. Department of Agriculture because of the superior flight ability of adult females compared to females of the European biotype. To identify genes that potentially account for differences in female flight capability between the two biotypes, we assembled and compared transcriptional profiles of two North American populations of ESM and two Chinese populations of ASM, including samples of unmated female adults and females after mating and oviposition. Of 129,286 unigenes identified, 306 were up-regulated in ASM samples relative to ESM, including genes involved in egg production. In contrast, 2309 unigenes were down-regulated in ASM samples, including genes involved in energy production. Although a previous study found that ASM female flight was reduced after oviposition, a comparison of gene expression before and after mating and oviposition did not reveal any genes which were consistently up- or down-regulated in the two ASM populations.

## Introduction

The spongy moth *Lymantria dispar* L., formerly known as the gypsy moth, is native to Europe and Asia. It is considered to be one of the most destructive forest defoliators over much of its range^1,2^. The spongy moth was accidentally introduced into North America in Medford, Massachusetts in the 1860s^3^. Since then, the spongy moth has spread throughout much of the northeastern seaboard of the United States and adjacent parts of Canada. Spongy moth larvae feed on more than 300 species of trees, causing defoliation in coniferous and deciduous forests as well as residential areas^4–7^ .

The spongy moth has been further classified by Pogue and Schaefer^8^ into three subspecies: the European subspecies *L. dispar dispar* L., the Asian subspecies *L. dispar asiatica* Vnukovskij, and the Japanese subspecies *L. dispar japonica* Motschulsky. For regulatory purposes, moths of the latter two subspecies are grouped into a biotype that had been formerly referred to as the Asian gypsy moth, along with *Lymantria umbrosa* and *Lymantria postalba*^9^^.^ This biotype is defined by the capacity of females to fly, in contrast to females of the European/North American spongy moth which are characterized by females that are largely flightless. This delineation of the spongy moth into subspecies and biotypes has been supported by comparative analyses of the mitochondrial genomes of different spongy moth populations and by a genotyping-by-sequencing analysis involving 2327 single-nucleotide polymorphisms, although these studies have also revealed differences among populations of a subspecies^10,11^. Subspecies of the Asian spongy moth biotype (hereafter abbreviated as ASM) are mainly distributed from the Ural Mountains east to China, South Korea, Japan, and the Russian Far East. The European spongy moth biotype (ESM) is distributed in Europe, east North America, west and central Asia, north Africa, north India, Pakistan and Afghanistan. Compared with ESM, some populations of ASM may also require a shorter time to break the diapause of its eggs^12^ and may be able to better adapt to some North American plants than the established ESM^13^. These properties suggest that ASM may cause more damage and loss than ESM if it becomes established in North America.

Female adult flight capability is the only criterion for classification of spongy moth populations as ASM^8^. While ESM females in North American populations are generally not capable of any kind of flight^14,15^, variability in female flight capability and activity has been observed among other strains of ESM from Europe and among populations of ASM^16–19^. Correlations between variations in female spongy moth flight capacity and variations in wing size and dimensions, flying muscle tissue, and wing load (mass/wing area) have been reported^15–17,20^.

Analysis of crosses between ASM and ESM moths indicate that flight capability has a significant genetic basis^14,15^. However, while broad geographic groups of *L. dispar* can be distinguished with mitochondrial and nuclear genetic markers^10^, alleles of these markers often were not completely fixed in regions where they occur and thus could not serve as unambiguous indicators of female flight capability^16,21,22^. In addition to biotype- and strain-dependent physiological factors influencing flight, mated ASM adult females were found to have significantly reduced flight capability after oviposition^23^, but not before^17^. Spongy moth adults are capital breeders that rely on resources accumulated as larvae to carry out flight and reproduction^24^. The effect of oviposition on ASM flight may represent the need for resorption of oocytes to supply fuel for prolonged flight activity, a resource that is lost upon oviposition.

A previous analysis and comparison of genomic sequence data derived from ESM and ASM samples detected genetic divergence between the two biotypes in a selection of genes enriched in gene ontology (GO) categories presumed to be involved in flight, such as “skeleton muscle adaptation” (GO:0043501) and “ionotropic glutamate receptors” (GO:0035235)^25^. Some of the divergent genes encoded homologs of genes that control wing size in *Drosophila melanogaster*. Results of this analysis also indicated that a greater degree of sequence divergence may exist in the regulatory regions of ESM and ASM genomes, suggesting that differences in gene expression may also contribute to differences in flight capacity. A comparative analysis of ESM and ASM female antennal and larval head capsule transcriptomes has been published which identified differences in olfaction-related gene expression among three representative strains of the two biotypes^26^, which is consistent with the concept that differences in gene expression may account for differences in flight capacity.

In this study, our goals were to test this concept and (1) identify key genes expressed in adult moths that potentially affect the flight ability and wing development of female spongy moth, (2) explore their expression characteristics and differences in distinct geographical populations, and (3) provide a basis for further research on the molecular mechanism of flight ability. We used transcriptome sequencing to analyze the female adults of two different populations each from the United States and China in order to identify differences in gene expression between ESM and ASM that are consistently observed. Eight libraries prepared from RNA harvested from adult females of these four strains before mating and after mating and oviposition were constructed and sequenced, and differentially expressed genes that might affect their flight activities were analyzed and assessed.

## Results

### Qualitative description for assembly and annotation of transcriptomes

We assembled and compared genome-wide transcription profiles of ESM and ASM virgin adult females and females after mating and oviposition. Eight independent RNA-Seq analyses were performed with different populations.

A total of 205.96 Gb of processed reads were obtained by sequencing (> 6.98 Gb/replicate). The percentage of Q30-filtered bases was more than 91.85%. Assembly of the sequence data from all libraries resulted in identification of 129,286 unigenes (Table 1).

**Table 1:** Transcriptome assembly results for all samples.

Type	Result
Total transcripts (filtered)	176,654
Total unigenes	129,286
Total sequence, bases	120,861,580
Unigene/transcript average length	684.17
E90N50	2400
GC percent	39.13
Mean mapped reads	3737.048531
TransRate score	0.15799
BUSCO score	C: 96.8% (S: 86.2%; D: 10.6%)

**Figure 1:**
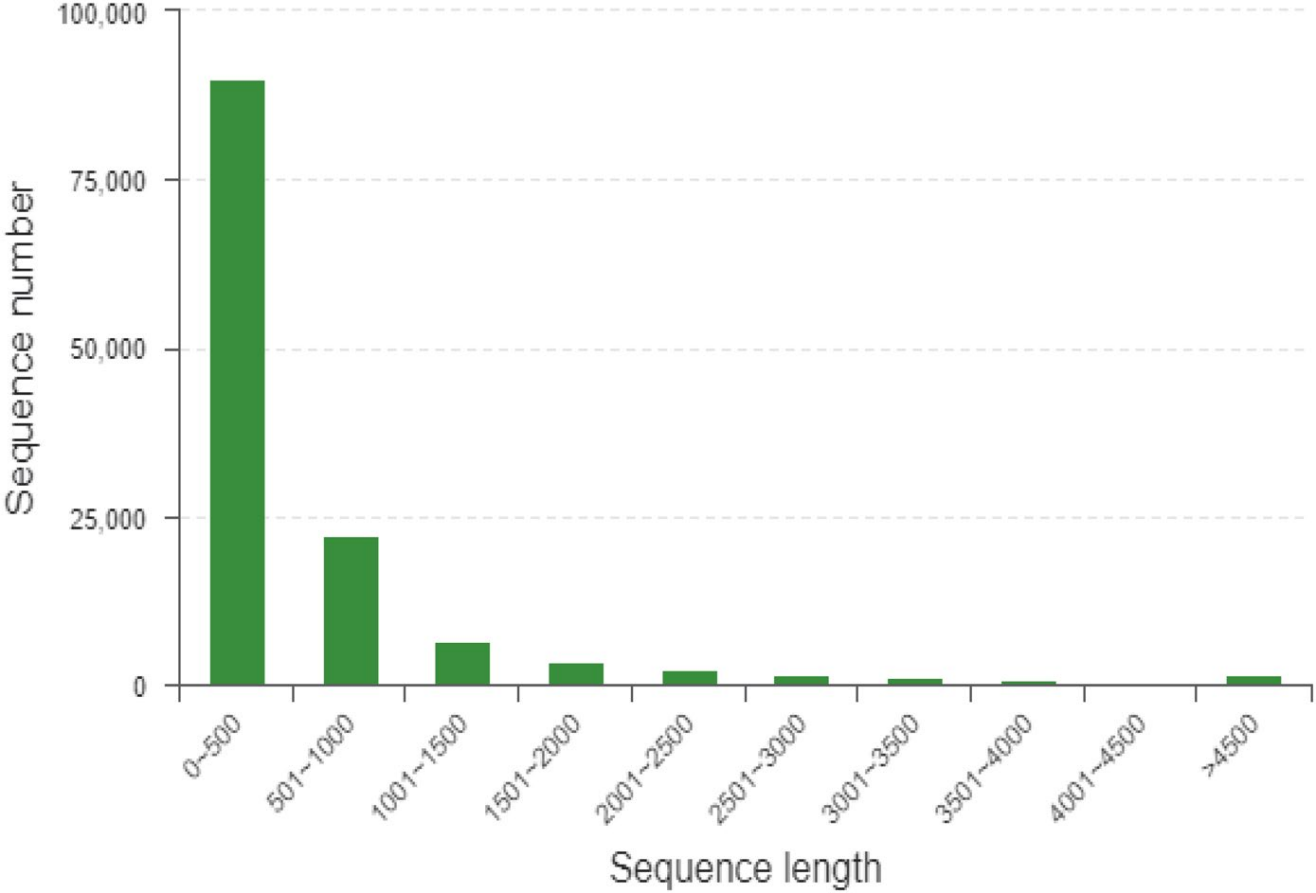
Length distribution of unigenes/transcripts among all spongy moth samples.

The length distribution of the unigenes is shown in Fig. 1. The percentage of unigenes with a length not greater than 500 bp was over 70%, with a further 17% ranging between 501 and 1000 bp. Relatively few unigenes were found in the 4001–4500 bp category.

All female adult transcriptome unigenes were annotated from the NR, Swiss-Prot, Pfam, COG, GO and KEGG databases. The number of unigene annotations was 39,584, accounting for 30.62% of the total. Among them, queries of the NR database yielded the most annotations, accounting for 26.28% of the total. The COG database yielded the least annotations, 6,221 (4.81%) (Table 2).

**Table 2:** Unigenes annotation profiles of all spongy moth samples.

Database	Unigene number (proportion)
NR	33,971 (0.2628)
Swiss-Prot	27,450 (0.2123)
Pfam	24,831 (0.1921)
COG	6221 (0.0481)
GO	22,408 (0.1733)
KEGG	21,260 (0.1644)
Total annotated	39,584 (0.3062)
Total	129,286 (1)

Total annotated = number of unigenes annotated in one or more databases.

### NR annotation

**Figure 2:**
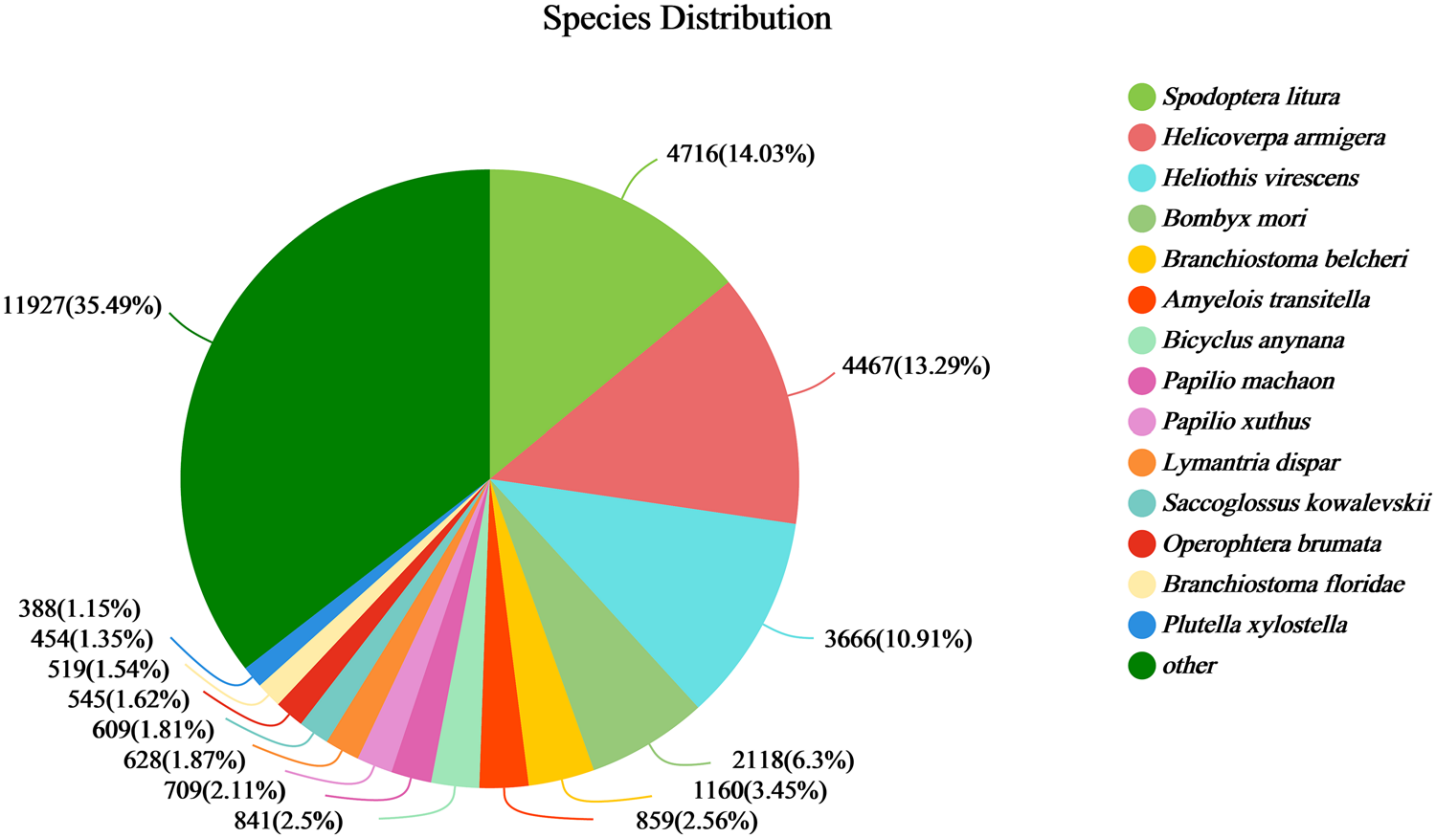
Species distribution of BLAST results from female spongy moth transcriptomes.

BLASTX comparison with NR database sequences was performed to identify the similarity between the transcription sequences of spongy moth and similar species and the functional information of homologous sequences. A total of 33,971 unigenes were successfully matched with known genes (E < 10^–5^), with 4716 (14.03%) sequences showing high sequence similarity with *Spodoptera litura*, followed by *Helicoverpa armigera*. There were 4467 (13.29%) and 3666 (10.91%) unigenes with top BLAST matches with *Heliothis virescens* sequences. Matches with other species returned by BLAST showed low sequence similarity with spongy moth sequences. However, 11,927 unigenes (35.49%) were unique transcripts of spongy moth (Fig. 2).

### COG/NOG annotations

**Figure 3:**
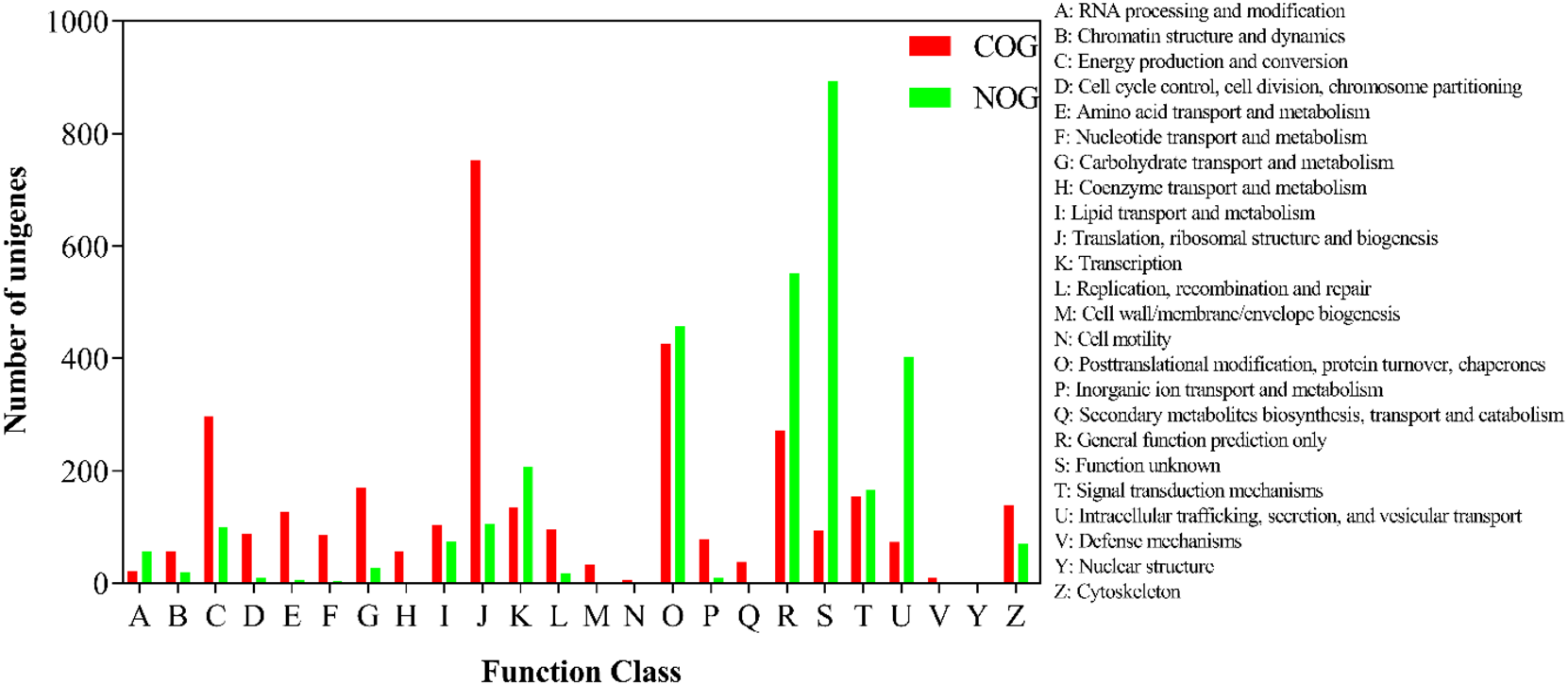
COG/NOG functional annotation of adult spongy moth transcriptomes.

COG (Clusters of Orthologous Groups) is a database of protein lineages for general function prediction, while NOG (Non-Supervised Orthologous Groups) is optimized on the basis of COG to expand genomic information and provide more detailed OG analysis. After comparison, COG function classification of genes/transcripts can be obtained. The most prevalent COG functions annotated in the transcriptomes of adult spongy moth are *translation, ribosomal structure and biogenesis*, while the *Function unknown* class was the most prevalent annotation in NOG (Fig. 3).

### GO annotation

**Figure 4:**
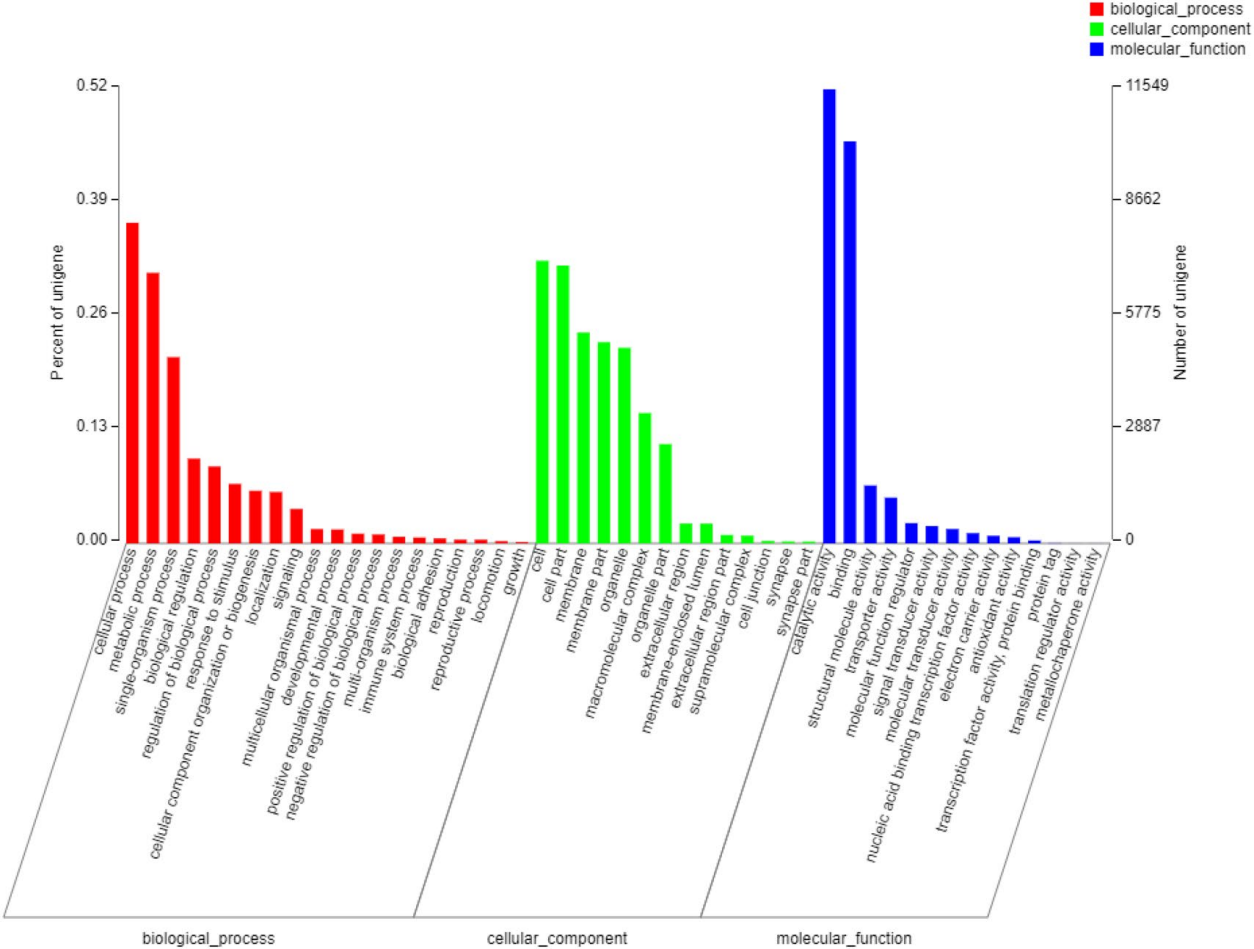
GO functional annotation of adult female spongy moth transcriptomes.

A total of 22,408 Unigene sequences were annotated with 94,399 GO entries, including 48 subclasses in 3 categories: 20 *biological_processes* subclasses, 14 *cell_components* subclasses, and 14 *molecular_functions* subclasses. The largest number of annotations were for *biological process* (37,112; 39.3%), and the least was *molecular_function* (26,424; 28%) (Fig. 4).

### KEGG pathway

**Figure 5:**
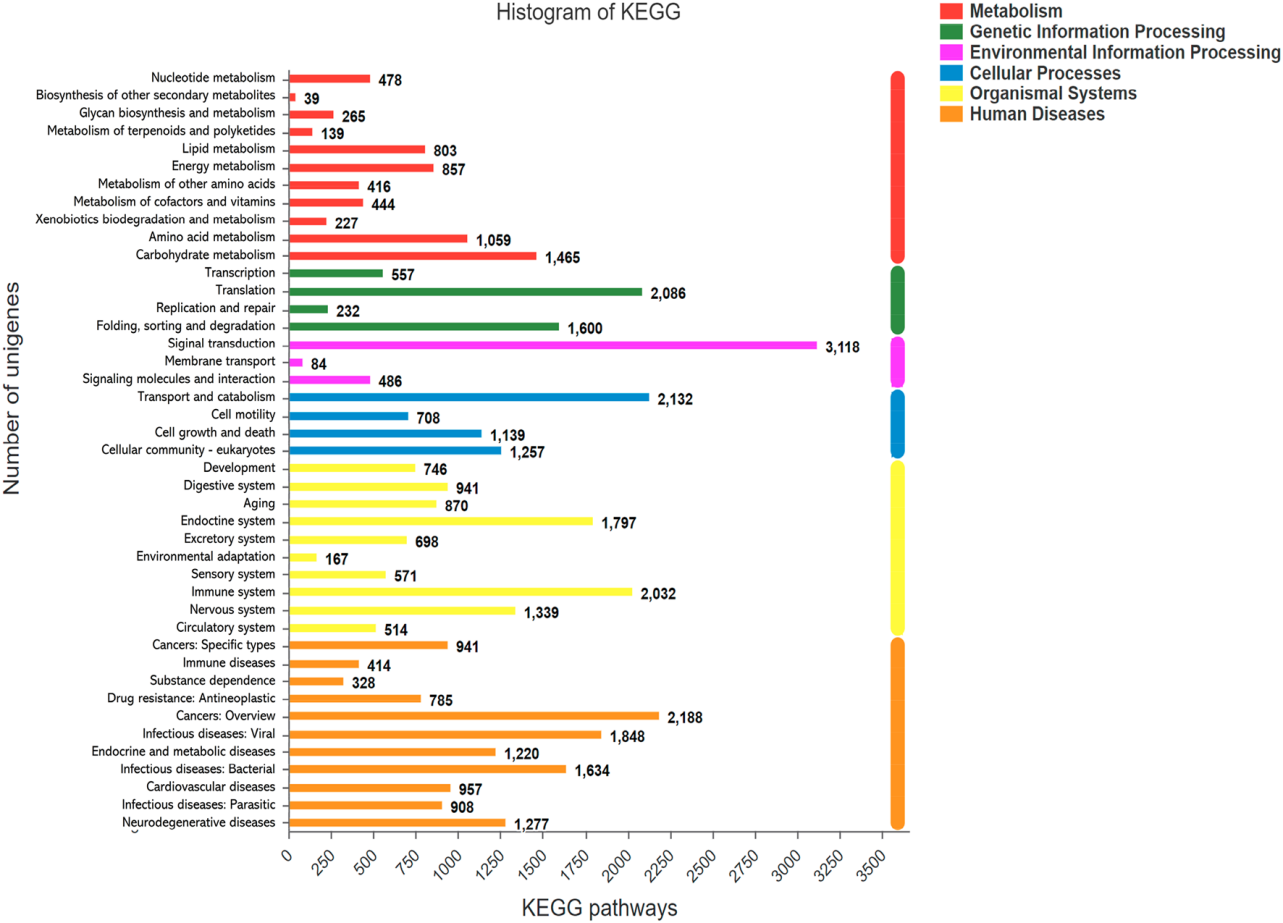
Mapping of KEGG pathway functions of transcripts in adult female spongy moth transcriptomes.

Biological functions of transcripts in the spongy moth transcriptomes were identified with the assistance of KEGG (Kyoto Encyclopedia of Genes and Genomes), a large knowledge base for analyzing gene functions and linking genomic information with functional information. We found that there was a total of 21,260 unigenes that mapped to six pathways, including *Metabolism*, *Genetic Information Processing*, *Environmental Information Processing*, *Cellular Processes*, *Organismal Systems*, and *Human Diseases*. Among them, the most unigenes (3118) mapped to the *Signal Transduction* subgroup under *Environmental Information Processing*^27–29^(Fig. 5).

### Strain-specific differences in gene expression

A total of 6692 unigenes were found to be differentially expressed in pairwise comparisons of ASM and ESM transcriptomes, including 5371 up-regulated and 1321 down-regulated DEGs in ASM relative to ESM (Supplementary Table 1). Approximately two orders of magnitude more differentially-expressed genes (DEGs) were observed in pairwise comparisons of ASM and ESM transcriptomes (JGS vs. CT, JGS vs. NJ, ZY vs. CT, ZY vs. NJ) relative to the number of DEGs found with pairwise comparisons of transcriptomes from the same biotype (JGS vs. ZY, CT vs. NJ). The number of the DEGs are shown in Table 3.

**Table 3:** The number of up-regulated and down-regulated DEGs between four geographic spongy moth strains.

Groups	Up-regulated^a^	Down-regulated^a^
JGS_ZY	108	53
CT_NJ	19	11
JGS_CT	3434	757
JGS_NJ	3027	592
ZY_CT	3201	888
ZY_NJ	2995	708

*JGS* Jingeshan, *ZY* Zunyi, *CT* Connecticut, *NJ* New Jersey.

^a^Numbers refer to the quantity of up- or down-regulated DEGs in the first strain listed in each pairwise comparison relative to the second strain.

In four pairwise comparisons of ASM transcriptomes with ESM transcriptomes (e.g., JGS vs CT, JGS vs NJ, ZY vs CT, and ZY vs NJ), 306 DEGs consistently exhibited up-regulation and 2,309 DEGs consistently exhibited down-regulation in ASM transcriptomes relative to ESM transcriptomes (Supplementary Table 2). Table 4 lists the 40 DEGs with the greatest degree of up- or down-regulation in ASM-ESM pairwise comparisons. The 20 DEGs with the greatest degree of down-regulation in ASM relative to ESM included cytochrome c oxidase (COX) subunits I, II & III; cytochrome b oxidase; NADH dehydrogenase subunits 1 & 4; ATP synthase subunit 6; glucose dehydrogenase; myelin protein P0 isoform L-MPZ precursor; myelin basic protein isoform X3; myosin-4; and moricin. Among the 20 DEGs up-regulated to the greatest extent in ASM transcriptomes were pancreatic triacylglycerol lipase-like, alkaline C trypsin, calphotin-like, serine protease 1-like, non-specific lipid-transfer protein, L-serine dehydratase/L-threonine deaminase, trypsin precursor AiT6, actin cytoskeleton-regulatory complex protein PAN1-like, NADH dehydrogenase subunit 1, vitellogenin 7 precursor, vitellogenin 2 isoform 1 precursor, and chitin deacetylase 1 & 8.

**Table 4:** Genes that were consistently differentially expressed in all comparisons in four ASM and ESM transcriptomes.

Gene ID	Comparison	log2FoldChange	Adj-P^30^	Gene expression level (GEL)				NR hits
				CN_JGS	CN_ZY	USA_CT	USA_NJ	
TRINITY_DN56697_c1_g2	ZY_NJ	–34.83374762	2.86E–56	–	0	–	67.14	None
	ZY_CT	−34.35069549	1.55E–54	–	0	70.09	–	
	JGS_NJ	−24.73745878	4.62E−27	0	–	–	67.14	
	JGS_CT	−24.25440666	5.39E−26	0	–	70.09	–	
TRINITY_DN13504_c0_g1	ZY_NJ	−34.17627032	9.52E−50	–	0	–	79	None
	ZY_CT	−34.26177166	5.12E−50	–	0	89.51	–	
	JGS_NJ	−24.49561347	1.83E−24	0	–	–	79	
	JGS_CT	−24.58111481	1.14E−24	0	–	89.51	–	
TRINITY_DN58366_c8_g2	ZY_NJ	−27.45125641	2.58E−08	–	0	–	0.37	XP_028035360.1 gloverin 4
	ZY_CT	−33.80514904	8.43E−13	–	0	21.88	–	
	JGS_NJ	−16.36806188	0.005321	0	–	–	0.37	
	JGS_CT	−22.7219545	8.32E−06	0	–	21.88	–	
TRINITY_DN33204_c1_g1	ZY_NJ	−27.85583552	1.27E−08	–	0	–	57.34	None
	ZY_CT	−29.99664328	4.52E−10	–	0	90.23	–	
	JGS_NJ	−18.12384237	0.001125	0	–	–	57.34	
	JGS_CT	−20.26465013	0.000121	0	–	90.23	–	
TRINITY_DN72186_c0_g1	ZY_NJ	−25.36839952	4.25E−10	–	0	–	2.65	AKJ54509.1 moricin
	ZY_CT	−31.15021215	1.26E−15	–	0	63.53	–	
	JGS_NJ	−16.27525422	0.000381	0	–	–	2.65	
	JGS_CT	−22.05706685	8.5E−08	0	–	63.53	–	
TRINITY_DN83020_c0_g1	ZY_NJ	−27.25754436	8.35E−10	–	0	–	5.9	None
	ZY_CT	−26.94823185	1.28E−09	–	0	2.84	–	
	JGS_NJ	−21.67189109	3.4E−06	0	–	–	5.9	
	JGS_CT	−21.36257859	4.68E−06	0	–	2.84	–	
TRINITY_DN61670_c0_g1	ZY_NJ	−25.33900066	4.94E−07	–	0	–	0.21	None
	ZY_CT	−31.4905229	4.2E−11	–	0	20.97	–	
	JGS_NJ	−15.54852005	0.010722	0	–	–	0.21	
	JGS_CT	−21.7000423	2.65E−05	0	–	20.97	–	
TRINITY_DN23060_c0_g1	ZY_NJ	−26.72574954	6.03E−10	–	0	–	3.73	XP_026735683.1 glucose dehydrogenase [FAD, quinone]-like
	ZY_CT	−26.19938465	1.36E−09	–	0	2.52	–	
	JGS_NJ	−21.551178	1.85E−06	0	–	–	3.73	
	JGS_CT	−21.02481311	3.5E−06	0	–	2.52	–	
TRINITY_DN38260_c0_g1	ZY_NJ	−9.16728883	2.38E−15	–	0	–	18.88	ABG11762.1 cytochrome b
	ZY_CT	−8.727985703	8.95E−14	–	0	15.11	–	
	JGS_NJ	−9.427663179	2.68E−16	0	–	–	18.88	
	JGS_CT	−8.988360052	1.12E−14	0	–	15.11	–	
TRINITY_DN42442_c0_g1	ZY_NJ	−9.226958661	8.6E−16	–	0	–	7.15	ABV22517.1 NADH dehydrogenase subunit 4
	ZY_CT	−8.750104651	4.76E−14	–	0	5.17	–	
	JGS_NJ	−9.487333085	9.01E−17	0	–	–	7.15	
	JGS_CT	−9.010479076	5.73E−15	0	–	5.17	–	
TRINITY_DN55289_c1_g2	ZY_NJ	−9.300640442	6.44E−16	–	0	–	3.69	YP_004111298.1 cytochrome c oxidase subunit I
	ZY_CT	−8.762913375	5.83E−14	–	0	2.64	–	
	JGS_NJ	−9.561014708	6.9E−17	0	–	–	3.69	
	JGS_CT	−9.023287641	7.15E−15	0	–	2.64	–	
TRINITY_DN45473_c0_g2	ZY_NJ	−9.265572917	3.12E−16	–	0	–	30.97	YP_009024856.1 cytochrome c oxidase subunit II
	ZY_CT	−8.815963642	1.53E−14	–	0	22.99	–	
	JGS_NJ	−9.525947428	3.29E−17	0	–	–	30.97	
	JGS_CT	−9.076338152	1.73E−15	0	–	22.99	–	
TRINITY_DN58407_c9_g1	ZY_NJ	−8.111566982	0.001375	–	0	–	34.14	CAB3230287.1 unnamed protein product
	ZY_CT	−10.14449048	9.24E−06	–	0	74.26	–	
	JGS_NJ	−8.371917541	0.00082	0	–	–	34.14	
	JGS_CT	−10.40484104	4.44E−06	0	–	74.26	–	
TRINITY_DN57574_c0_g1	ZY_NJ	−9.372862201	3.12E−16	–	0	–	15.57	ACP50397.1 NADH dehydrogenase subunit 1
	ZY_CT	−8.890982681	1.89E−14	–	0	12.23	–	
	JGS_NJ	−9.633236329	3.34E−17	0	–	–	15.57	
	JGS_CT	−9.151356809	2.23E−15	0	–	12.23	–	
TRINITY_DN81391_c0_g1	ZY_NJ	−9.515567545	5.48E−17	–	0	–	7.7	NP_001300997.1 myelin protein P0 isoform L-MPZ precursor
	ZY_CT	−8.805274946	2.58E−14	–	0	4.96	–	
	JGS_NJ	−9.775941742	5.36E−18	0	–	–	7.7	
	JGS_CT	−9.065649142	3.05E−15	0	–	4.96	–	
TRINITY_DN56710_c3_g2	ZY_NJ	−9.407242996	3.66E−15	–	0	–	23.56	None
	ZY_CT	−9.165370434	2.58E−14	–	0	22.13	–	
	JGS_NJ	−9.667616094	4.54E−16	0	–	–	23.56	
	JGS_CT	−9.425743532	3.38E−15	0	–	22.13	–	
TRINITY_DN38334_c0_g1	ZY_NJ	−10.16900024	2.33E−19	–	0	–	41.75	AAD15020.1 cytochrome oxidase III
	ZY_CT	−9.685566124	2.08E−17	–	0	31.08	–	
	JGS_NJ	−10.42937307	1.82E−20	0.17	–	–	41.75	
	JGS_CT	−9.945938954	1.98E−18	0.17	–	31.08	–	
TRINITY_DN43889_c0_g1	ZY_NJ	−10.24363704	3.43E−20	–	0	–	37.25	ABG11760.1 ATP synthase F0 subunit 6
	ZY_CT	−9.726532977	4.72E−18	–	0	27.24	–	
	JGS_NJ	−10.50401005	2.29E−21	0.14	–	–	37.25	
	JGS_CT	−9.98690599	4.23E−19	0.14	–	27.24	–	
TRINITY_DN24402_c0_g1	ZY_NJ	−10.26592127	3.66E−20	–	0	–	11.26	XP_006255059.1 myelin basic protein isoform X3
	ZY_CT	−9.868460973	1.73E−18	–	0	8.79	–	
	JGS_NJ	−10.52629408	2.47E−21	0	–	–	11.26	
	JGS_CT	−10.12883379	1.39E−19	0	–	8.79	–	
TRINITY_DN58997_c0_g1	ZY_NJ	−9.162508683	2.23E−15	–	0	–	1.57	NP_062198.1 myosin-4
	ZY_CT	−8.698819402	1.02E−13	–	0	1.15	–	
	JGS_NJ	−9.422883087	2.5E−16	0	–	–	1.57	
	JGS_CT	−8.959193806	1.29E−14	0	–	1.15	–	
TRINITY_DN59309_c7_g2	ZY_NJ	11.83843455	5.95E−19	–	22.91	–	0	VDM16675.1 unnamed protein product
	ZY_CT	11.95616158	2.34E−19	–	22.91	0	–	
	JGS_NJ	14.11976023	9.51E−28	113.37	–	–	0	
	JGS_CT	14.23748726	2.87E−28	113.37	–	0	–	
TRINITY_DN59741_c2_g1	ZY_NJ	12.72258468	5.81E−34	–	41.06	–	0	NP_001096141.1 vitellogenin 7 precursor
	ZY_CT	12.84031173	1.24E−34	–	41.06	0	–	
	JGS_NJ	12.27073794	4.19E−31	27.32	–	–	0	
	JGS_CT	12.388465	6.82E−32	27.32	–	0	–	
TRINITY_DN36137_c0_g1	ZY_NJ	12.69990581	8.59E−27	–	10.56	–	0	None
	ZY_CT	12.81763286	2.7E−27	–	10.56	0	–	
	JGS_NJ	12.11782678	3.91E−24	118.37	–	–	0	
	JGS_CT	12.23555382	1.14E−24	118.37	–	0	–	
TRINITY_DN48344_c0_g2	ZY_NJ	10.1051722	3.12E−15	–	19.64	–	0	XP_012454349.1 PREDICTED: non-specific lipid-transfer protein
	ZY_CT	10.22289924	1.26E−15	–	19.64	0	–	
	JGS_NJ	11.20217362	4.63E−19	40.95	–	–	0	
	JGS_CT	11.31990065	1.6E−19	40.95	–	0	–	
TRINITY_DN55003_c0_g1	ZY_NJ	12.14483652	1.41E−31	–	45.21	–	0	XP_037298773.1 trypsin, alkaline C
	ZY_CT	12.26256357	3.02E−32	–	45.21	0	–	
	JGS_NJ	11.9242181	3.65E−30	38.57	–	–	0	
	JGS_CT	12.04194515	6.18E−31	38.57	–	0	–	
TRINITY_DN52890_c0_g1	ZY_NJ	12.04554237	5.43E−32	–	35.1	–	0	XP_026733233.1 pancreatic triacylglycerol lipase-like
	ZY_CT	12.16326943	1.14E−32	–	35.1	0	–	
	JGS_NJ	11.83092459	1.43E−30	28.71	–	–	0	
	JGS_CT	11.94865165	2.3E−31	28.71	–	0	–	
TRINITY_DN55622_c0_g1	ZY_NJ	11.88970792	3.8E−30	–	25.76	–	0	ACD37362.1 chitin deacetylase 1
	ZY_CT	12.00743497	7.62E−31	–	25.76	0	–	
	JGS_NJ	11.66997913	7.92E−29	20.94	–	–	0	
	JGS_CT	11.78770619	1.73E−29	20.94	–	0	–	
TRINITY_DN42377_c0_g1	ZY_NJ	11.28750003	3.7E−23	–	4.54	–	0	KAF4015244.1 hypothetical protein G4228_006027
	ZY_CT	11.40522708	1.09E−23	–	4.54	0	–	
	JGS_NJ	11.55074074	2.75E−24	5.09	–	–	0	
	JGS_CT	11.66846779	7.82E−25	5.09	–	0	–	
TRINITY_DN50963_c0_g2	ZY_NJ	10.48784096	7.27E−20	–	4.55	–	0	ABV70868.1 NADH dehydrogenase subunit 1
	ZY_CT	10.60556801	2.49E−20	–	4.55	0.02	–	
	JGS_NJ	10.94915507	7.24E−22	5.84	–	–	0	
	JGS_CT	11.06688212	2.03E−22	5.84	–	0.02	–	
TRINITY_DN57295_c1_g1	ZY_NJ	11.44001779	1.54E−27	–	24.35	–	0	XP_021183170.1 calphotin-like
	ZY_CT	11.55774485	3.55E−28	–	24.35	0	–	
	JGS_NJ	11.14731591	5.82E−26	19.31	–	–	0	
	JGS_CT	11.26504296	1.4E−26	19.31	–	0	–	
TRINITY_DN56232_c0_g2	ZY_NJ	11.41968572	6.3E−28	–	19.12	–	0	XP_026746126.1 serine protease 1-like
	ZY_CT	11.53741278	1.42E−28	–	19.12	0	–	
	JGS_NJ	11.11378986	2.96E−26	15.33	–	–	0	
	JGS_CT	11.23151692	7.04E−27	15.33	–	0	–	
TRINITY_DN50167_c0_g1	ZY_NJ	10.91821699	7.75E−20	–	4.7	–	0	NP_001069130.1 L-serine dehydratase/L-threonine deaminase
	ZY_CT	11.03594403	2.79E−20	–	4.7	0	–	
	JGS_NJ	11.11276535	1.31E−20	5	–	–	0	
	JGS_CT	11.2304924	4.31E−21	5	–	0	–	
TRINITY_DN59741_c3_g2	ZY_NJ	11.34624617	6.95E−25	–	20.09	–	0	NP_001038378.1 vitellogenin 2 isoform 1 precursor
	ZY_CT	11.46397323	1.94E−25	–	20.09	0	–	
	JGS_NJ	10.59073727	2.52E−21	11.71	–	–	0	
	JGS_CT	10.70846432	7.7E−22	11.71	–	0	–	
TRINITY_DN40081_c0_g1	ZY_NJ	11.06828843	1.34E−26	–	16.58	–	0	XP_021200296.1 pancreatic triacylglycerol lipase-like
	ZY_CT	11.18601549	3.38E−27	–	16.58	0	–	
	JGS_NJ	10.86791667	1.85E−25	13.61	–	–	0	
	JGS_CT	10.98564372	4.61E−26	13.61	–	0	–	
TRINITY_DN45101_c0_g1	ZY_NJ	11.02602771	1.13E−26	–	14.45	–	0	XP_014365314.2 chitin deacetylase 8
	ZY_CT	11.14375477	2.83E−27	–	14.45	0	–	
	JGS_NJ	10.71648951	5.86E−25	11.31	–	–	0	
	JGS_CT	10.83421657	1.38E−25	11.31	–	0	–	
TRINITY_DN47054_c0_g1	ZY_NJ	10.98642411	2.48E−25	–	23.55	–	0	AAF74732.1 trypsin precursor AiT6
	ZY_CT	11.10415117	6.86E−26	–	23.55	0	–	
	JGS_NJ	10.68823818	8.35E−24	18.27	–	–	0	
	JGS_CT	10.80596523	2.05E−24	18.27	–	0	–	
TRINITY_DN57295_c1_g2	ZY_NJ	10.96724498	3.16E−25	–	39.86	–	0	XP_022821848.1 actin cytoskeleton-regulatory complex protein PAN1-like
	ZY_CT	11.08497204	8.7E−26	–	39.86	0	–	
	JGS_NJ	10.69980682	7.81E−24	31.87	–	–	0	
	JGS_CT	10.81753387	1.92E−24	31.87	–	0	–	
TRINITY_DN47254_c0_g1	ZY_NJ	10.99043276	8.09E−25	–	10.79	–	0	XP_021326591.1 vitellogenin-like
	ZY_CT	11.10815982	2.15E−25	–	10.79	0	–	
	JGS_NJ	10.61636625	6.78E−23	7.69	–	–	0	
	JGS_CT	10.7340933	1.61E−23	7.69	–	0	–	
TRINITY_DN57949_c0_g1	ZY_NJ	9.429327246	1.42E−11	–	2.38	–	0	XP_019817766.1 uncharacterized protein LOC109560216 isoform X1
	ZY_CT	9.547054274	6.22E−12	–	2.38	0	–	
	JGS_NJ	11.7584304	8.97E−19	12.24	–	–	0	
	JGS_CT	11.87615743	3.32E−19	12.24	–	0	–	
TRINITY_DN47831_c0_g1	ZY_NJ	10.27760237	2.64E−17	–	8.85	–	0	AAY43793.1 E6-4
	ZY_CT	10.39532941	9E−18	–	8.85	0	–	
	JGS_NJ	11.09921467	1.63E−20	14.72	–	–	0	
	JGS_CT	11.21694171	5.24E−21	14.72	–	0	–	

DEGs are sorted in descending order with respect to log2-fold change, with down-regulated DEGs listed first, followed by up-regulated DEGs.

### Within-population differences in gene expression before mating and after mating and oviposition

Because adult female spongy moth flight was found to be significantly reduced after oviposition^23^, we also prepared transcriptomes from RNA of virgin adult females before mating and adult females after mating and oviposition and examined differences in gene expression. Table 5 shows the number of DEGs up-regulated and down-regulated in moths before mating relative to after mating and oviposition for all four strains. Noticeably more DEGs were identified for the ESM strains compared to the ASM strains.

**Table 5:** The number of DEGs up-regulated and down-regulated before mating (BM) relative to after mating and oviposition (AM).

Comparisons	Up-regulated^a^	Down-regulated^a^
JGS_BM-AM	18	7
ZY_BM-AM	3	4
CT_BM-AM	128	23
NJ_BM-AM	592	21

^a^Numbers refer to the quantity of up- or down-regulated DEGs in the before-mating (BM) sample relative to the after-mating/oviposition (AM) sample of each pairwise comparison.

No DEGs were identified that were consistently up-regulated or down-regulated in comparisons of the before-mating and after-mating and oviposition of both ASM strains. In contrast, 58 DEGs were found to be consistently up-regulated or down-regulated in these comparisons for the two ESM strains (Supplementary Table 2). All but one of these were up-regulated in the before-mating transcriptomes. Table 6 lists the single DEG which was down-regulated in ESM before-mating transcriptomes and the ten DEGs up-regulated in before-mating transcriptomes with the greatest degree of difference. The up-regulated DEGs included lysocardiolipin acyltransferase 1-like and actin muscle-type A2. One of the up-regulated DEGs in ESM comparisons was also found to be up-regulated in NJ before-mating transcriptomes (Table 5, TRINITY_DN53843_c6_g1).

**Table 6:** Genes that were consistently differentially expressed (DEGs) in all comparisons of ASM and ESM transcriptomes developed from moths before mating (BM) and after mating and oviposition (AM).

Gene ID	Comparison	log2FoldChange	Adjp	GEL			NR hits
				BM		AM	
TRINITY_DN55444_c2_g1	NJ_BM-AM	−41.9624	2.31E−50	0		6.16	None
	JGS_BM-AM	−14.4299	0.005106	0		0.5	
TRINITY_DN1848_c0_g1	NJ_BM-AM	24.30176	5.84E−05	5.64		0	KAF9410092.1 hypothetical protein HW555_010723, partial
	CT_BM-AM	19.79454	0.01	0.19		0	
TRINITY_DN43723_c1_g1	NJ_BM-AM	9.462868	0.001434	17.64		0	XP_026734001.1 lysocardiolipin acyltransferase 1-like
	CT_BM-AM	17.25244	1.29E−09	0.11		0	
TRINITY_DN52100_c0_g1	NJ_BM-AM	10.50207	1.74E−05	7.02		0.03	CAB3223558.1 unnamed protein product
	CT_BM-AM	10.58316	2.39E−08	47.69		0.095	
TRINITY_DN59230_c4_g1	NJ_BM-AM	10.79498	0.000204	31.28		0.13	NP_001119725.1 actin, muscle-type A2
	CT_BM-AM	9.604437	0.000516	69.35		0.27	
TRINITY_DN46116_c0_g1	NJ_BM-AM	10.90386	6.46E−06	25.56		0.1	CAB3226619.1 unnamed protein product
	CT_BM-AM	7.596039	0.000876	24.06		0.44	
TRINITY_DN47299_c0_g1	NJ_BM-AM	10.29331	4.96E−08	119.68		0.3	KAG8112956.1 hypothetical protein SFRUCORN_010746
	CT_BM-AM	7.629028	0.000988	136.95		2.28	
TRINITY_DN24521_c0_g1	NJ_BM-AM	7.680281	0.001124	25.85		0.41	CAB3226695.1 unnamed protein product
	CT_BM-AM	9.814032	0.000842	9.21		0	
TRINITY_DN59230_c7_g1	NJ_BM-AM	7.093234	0.036808	123.95		0	AEB26312.1 actin
	CT_BM-AM	9.75179	0.001983	82.18		0	
TRINITY_DN40225_c0_g1	NJ_BM-AM	7.894189	0.001201	6.04		0.17	CAB3239390.1 unnamed protein product
	CT_BM-AM	7.019374	0.005982	10.56		0.24	
TRINITY_DN53843_c6_g1	NJ_BM-AM	4.759893	1.46E−05	1512.87		126.8	CAB3228006.1 unnamed protein product
	CT_BM-AM	4.407292	0.000516	780.49		72.87	
	JGS_BM-AM	4.014438	0.02672	715.44		41.71	

DEGs are sorted in descending order with respect to log2-fold change, with DEGs down-regulated in before-mating ESM transcriptomes listed first, followed by DEGs up-regulated in ESM before-mating transcriptomes.

## Discussion

The *L. dispar* genome, at approximately 1.0 Gb^31,32^, dwarfs the majority of sequenced Lepidoptera genomes that, on average, range from 250 to 500 Mb in size^25^. Genetic analysis has shown that ASM populations are more genetically diverse than ESM populations^33^. These features pose challenges to the identification of genes whose expression may account for the distinctive flightworthiness of ASM females. In an attempt to overcome these challenges, we carried out a comparative analysis among transcriptomes developed from adult females of two different ASM populations as well as two different North American ESM populations, and also included a comparative analysis of transcriptomes from virgin females and mated females after oviposition. Comparisons of differentially expressed genes identified genes which were consistently up- or down-regulated in adult ASM RNA samples. Some of the DEGs with the greatest differences in expression level between ASM and ESM populations (Table 4) appeared to bear some relevance to aspects of flight activity, such as flight muscle function and energy production. These DEGS included the following:

### Cytochrome oxidase (COX) subunits I, II, and III, and cytochrome b (CytB) (TRINITY_DN55289_c1_g2, TRINITY_DN45473_c0_g2, TRINITY_DN38334_c0_g1, and TRINITY_DN38260_c0_g1, respectively; Table 4*)*

Cythochromes were independently discovered in insect systems by Charles MacMunn and David Keilin. Keilin observed that “among all organisms examined, the highest concentration of cytochrome is found in the thoracic muscles of flying insects”^34,35^. Indeed, the high amount of cytochromes in insect flying muscle suggests that they play a role in biological oxidation and energy transmission, which is consistent with the large energy demand that flight activity places on this special tissue^36,37^. In mosquitoes, reductions in flight muscle mitochondrial metabolism triggered by a blood meal would lead to directing spare nutrients from flight muscle to ovaries in support of oogenesis^38,39^.This is part of the "flight-oogenesis syndrome," a physiological process in which some migratory insects switch between two energy-intensive states: migration and reproduction^40^. Evidence shows that flight metabolism and dispersal potential are tightly linked to cytochrome oxidase (COX) function. For example, long-distance migratory butterfly species have higher COX content and activity than short-distance fliers, and recently established populations of *Melitaea cinxia* butterflies have higher COX activity and dispersal potential than old ones^41^. This means that the relationship between dispersal potential and COX activity can also be observed within the same flying insect species. Given these observations, the finding that cytochrome b (CytB) and COX subunit I, II, and III DEGs were significantly down-regulated in ASM compared to ESM appears counterintuitive. However, mitochondrial COX genes showed evidence of relaxed selection in flightless as compared with flying lineages, as demonstrated by significantly higher dN/dS ratios in flightless lineages^42^. If there is also a lack of purifying selection pressure on the COX and CytB alleles of ESM, then the relevance of up-regulation of these genes for spongy moth female flight is unclear.

### Vitellogenin 7 precursor (TRINITY_DN59741_c2_g1), vitellogenin 2 isoform 1 precursor (TRINITY_DN59741_c3_g2), vitellogenin-like (TRINITY_DN47254_c0_g1)

In many oviparous species, vitellogenin (Vg) is a crucial precursor protein of egg yolk vitellin (Vn)^43^, which acts as an energy store. Vg is involved in oocyte maturation and development, making it a crucial protein involved in insect reproduction. A study of *Harmonia axyridis* reveals that Vg expression leads to increased egg production^44^. Three of the most up-regulated DEGs in ASM relative to ESM matched with Vg genes. While vitellogenin synthesis occurs primarily during the last larval instar in spongy moth, a Northern blot study detected relatively small steady-state quantities of vitellogenin RNA in adults, suggesting that completion of oogenesis initiated during the larval and pupal stages may require some small degree of vitellogenin synthesis early during the adult stage^45^. It is generally accepted that female spongy moths produce 500–1000 eggs, but there are no data indicating a consistently significant difference in egg production between ASM and ESM. There has been no formal study on the applicability of flight-oogenesis syndrome to ASM females, but some recent research suggests that there is not always an obvious trade-off between insect migratory flight and reproduction^46^.

### NADH dehydrogenase subunit 1 (TRINITY_DN57574_c0_g1, TRINITY_DN50963_c0_g2), NADH dehydrogenase subunit 4 (TRINITY_DN42442_c0_g1)

NADH dehydrogenase is involved in aerobic respiration and ATP synthesis^47^. Solitary locusts have higher initial flight speeds and shorter flight distances than gregarious locusts, and exhibited higher mitochondrial energetic storage (Acetyl-CoA and NADH), energy metabolic gene-expression levels, and metabolic enzyme activities in their flight muscles than their gregarious counterparts^48^. While NADH dehydrogenase subunit DEGs (for subunits 1 and 4) were found to be down-regulated in ASM, one DEG with matches to different NADH dehydrogenase subunit 1 sequences were up-regulated in ASM, suggesting either sequence divergence at this locus or two alleles with biotype-specific differences in their regulation.

### Myosin (TRINITY_DN58997_c0_g1, TRINITY_DN100147_c0_g1, TRINITY_DN22477_c0_g1, etc.), Actin (TRINITY_DN6120_c0_g1, TRINITY_DN59230_c5_g1, TRINITY_DN47814_c0_g1, etc.)

Actin, a filamentous protein (42 kD) involved in muscle contraction in both smooth and striated muscle, also serves as an important structural molecule for the cytoskeleton of many eukaryotic cells. It is the main constituent of the thin filaments of muscle fibers. Actin participates in many important cellular processes, including muscle contraction, cell motility, cell division and cytokinesis, vesicle and organelle movement, cell signaling, and the establishment and maintenance of cell junctions and cell shape. Actin filaments, usually in association with myosin, are responsible for many types of cell movements. Myosin is a type of molecular motor and converts chemical energy released from ATP into mechanical energy. This mechanical energy is then used to pull the actin filaments along, causing muscle fibers to contract and, thus, generating movement^49^. Actin and myosin are found in every type of muscle tissue. Thick myosin filaments and thin actin filaments work together to generate muscle contractions and movement. We found multiple myosin and actin genes down-regulated in ASM relative to ESM (Supplementary Table 2). Given that ASM female adults have strong flight ability, while ESM has no flight ability, the relevance of higher expression of these genes in ESM for spongy moth female flight is unclear. In DEGs compared within ESM populations, some actin genes were also found to be significantly higher before than after mating, which may reflect reduced muscle activity after mating.

### Comparison of gene expression before mating and after oviposition

Since ASM females are the ones in this study that are flightworthy, it was anticipated that meaningful differences in expression of genes before mating and after mating and oviposition would be observed in the transcriptomes of the JGS and ZY ASM strains and not in the flightless CT and NJ strains. However, few DEGs were identified in comparisons of ASM transcriptomes before mating and after oviposition, and no DEGs were found to be consistently up- or down-regulated in comparisons of these ASM transcriptomes. Thus, the results suggest that gene expression differences might not be the principal basis for the reported reduction in flight capacity of ASM females after oviposition^17^. It is interesting that comparisons of ESM transcriptomes before mating and after oviposition disclosed many more DEGs than the corresponding ASM comparisons, though the significance of this is unclear.

In conclusion, the results in this paper represent the first transcriptomic examination of gene expression in adult spongy moths. While DEGs with functions relevant to moth flight activity were identified in adult ASM and ESM transcriptomes, the trends in the differences in expression of these genes did not appear to be consistent with the differences in flight capabilities of ASM and ESM. DEGs expected to be up-regulated in flight-worthy *L. dispar* strains (such as cytochrome oxidase subunits, NADH dehydrogenase subunits, myosins and actins) often were found to be down-regulated instead. These results may reflect the possibility that the differences in gene expression relevant to female flight were subtle and hard to detect under the conditions the adults were sampled. Alternatively, differences in gene expression in ASM and ESM adults may affect flight capability by an unknown mechanism. It may also be the case that differences in gene expression of direct relevance for flight capability do not occur in adults of ASM and ESM. In addition, an examination of DEGs in adult females before mating and after mating and oviposition yielded no clues for why ASM female flight is reduced after mating and oviposition, but did reveal significant differences in gene expression before mating and after oviposition among ESM adults of two populations.

In a previous study, adult female wing size and wing load (body mass/wing area) were found to differ significantly among geographic strains of different biotypes as well as the same biotype, with larger wing sizes and lower wing loads observed in strains with greater flight capability^20^. This observation suggests that differences in the expression of genes controlling wing and body morphogenesis during development may account for strain-specific flight capability. A comparison of pupal transcriptomes may reveal differences in transcription during wing and wing muscle development in the pupal stage that may help to unlock the mystery of differential flight ability among the two spongy moth biotypes.

## Materials and methods

### Insect materials and RNA extraction

We analyzed four strains of *Lymantria dispar* from colonies derived from different geographic populations. Specimens of two ASM strains (*L. dispar asiatica*) from China were obtained from the Plant Quarantine Laboratory of Beijing Forestry University in 2019, the egg masses of JGS and ZY are collected in the wild from host trees, usually larches. After being brought back to the lab, then reared on artificial diet until pupation. Specimens of two ESM strains (*L. dispar dispar*) were obtained from the U.S. Department of Agriculture Animal and Plant Health Inspection Service Plant Protection and Quarantine^50^ program. Samples were processed at the Beijing Forestry University Plant Quarantine Laboratory in 2021 (Table 7). Eggs of all strains were hatched and the larvae reared on an artificial diet in a greenhouse under controlled conditions (temperature: 28 ± 0.5 °C)^51^. For pre-mating RNA samples, male and female pupae were separated prior to eclosion, and virgin females were harvested 24 h after hatching, because flight activity peaked when females were one day old and decreased thereafter. For post-oviposition RNA samples, females were mated with males and harvested within one hr after oviposition to avoid post-spawning mortality. All the samples were frozen in liquid nitrogen and immediately stored at -80℃.

**Table 7:** Location information for the four sampling sites of the spongy moth, *Lymantria dispar.*

Population code	Sampling Location	Latitude	Longitude
JGS	Jingeshan, Hebei, China	41°00′N	115°52′E
ZY	Zunyi, Guizhou, China	27°42′N	106°56’E
CT	Connecticut, USA	41°37′N	72°41′W
NJ	New Jersey, USA (New Jersey Standard Strain)	39.41′N	75.45′W

### RNA-Seq

RNA-seq data were generated from three biological replicates (five specimens/replicate) for each *L. dispar* strain, pre-mating and post-oviposition. Moths of each replicate were homogenized separately in two 2.0 mL tubes containing Lysing Matrix A (MP Biomedicals, Solon, OH, USA) and Lysis/Binding Solution from the mirVana™ miRNA Isolation Kit (ThermoFisher Scientific, Waltham, MA, USA) using a FastPrep-24™ Tissue and Cell Homogenizer (MP Biomedicals, Solon, OH, USA) set at 4.0 m/s and run for 40 s. Insoluble material was pelleted by centrifugation, and total RNA was recovered from the supernatants with the mirVana™ miRNA Isolation Kit. The RNA obtained was treated with DNase I (Invitrogen), and magnetic beads with oligo (dT) were used to isolate poly(A) + messenger RNA (mRNA), which was sheared into short fragments using a fragmentation buffer. Under the action of reverse transcriptase, six-base random primers (random hexamers) were added to synthesize one-stranded cDNA using mRNA as a template, followed by two-stranded synthesis to form a stable double-stranded structure. Termini of the double-stranded cDNA structure were blunted, and a terminal adenosine was added to the 3' end to facilitate library construction. The samples were submitted to the Majorbio Technologies company (Beijing, China) for quality assessment, construction of a non-stranded library using random hexamer priming, and 2 × 150 bp paired-end sequencing on an Illumina Novaseq 6000 instrument. RNA-seq data are available at National Center for Biotechnology Information (NCBI) Sequence Read Archives (SRA) under Bioproject accession numbers PRJNA789495 and PRJNA788963.

### Assembly and annotation of the transcriptome

Trinity (v2.13.2)^52^ was used for initial de novo assembly of Illumina sequence reads. The results of assembly with Trinity were then optimized for filtering and re-evaluated using TransRate (v1.0.3)^53^, CD-HIT (v4.8.1)^54^, and BUSCO (v5.2.2, using the arthropoda_odb10 database)^55^. The TGI Clustering Tool (v2.1) was employed to assemble the transcripts into unigenes^56^. The unigene assembly set is publicly available at the Open Science Framework (OSF) repository at (https://doi.org/10.17605/OSF.IO/PME7K). All unigenes obtained were compared with six databases (NR, Swiss-PROT, Pfam, COG, GO and KEGG databases) to provide annotation for the sequences in each database, and the annotation of each database was statistically analyzed. All unigenes were searched against these databases using BLAST (ftp://ftp.ncbi.nlm.nih.gov/blast/executables/blast+/2.2.29/) (e-value < 10^−5^). Protein function was predicted according to the most similar proteins annotated in these databases. Principle Components Analysis (PCA) was performed using the built-in prcomp function of R, which uses singular value decomposition, generally providing better numerical accuracy.

### Differential gene expression analysis

Differentially expressed genes (DEGs) were identified using DESeq2 (v1.18.1)^57^ together with salmon (v0.11.3), a read abundance quantification tool, operating in its quasi-alignment mode^58^. The R package, tximport (v1.20.0)^59^, was used to prepare counts at the gene-level as a function of transcript-level counts. Differential analysis was performed on these inputs using DESeq2’s DESeq function. Genes determined by salmon to have non-zero expression levels were flagged as differentially expressed by DESeq if they exhibited at least a two-fold difference in expression levels between the statistical factors being compared; furthermore, these were required to exhibit an adjusted p-value of 0.05 or less (alpha = 0.05, lfcThreshold = log2(2), altHypothesis = “greaterAbs”). Expression was also estimated at both the transcript and gene levels with RSEM (v1.2.24)^60^ using results from the bowtie2 short read aligner (v2.3.4.1)^61^ as input. RSEM-estimated abundances were expressed using the transcripts per million measure (TPM)^62^. Transcript sequences were aligned against the 2 February 2022 version of the NCBI NR protein database using DIAMOND (v0.9.22)^63^ in its BLASTX-like mode with default parameter settings. The top hit per query, if any, was recorded-if multiple best-scoring hits were encountered, a representative match was arbitrarily selected.

## Supplementary Information

Supplementary Table 1.

Supplementary Table 2.

## Data Availability

All data has been deposited at NCBI as indicated in the Methods. The datasets generated during and/or analyzed during the current study are available from the corresponding author on request.
